# Intestinal preservation in a birdlike dinosaur supports conservatism in digestive canal evolution among theropods

**DOI:** 10.1038/s41598-022-24602-x

**Published:** 2022-11-19

**Authors:** Xuri Wang, Andrea Cau, Bin Guo, Feimin Ma, Gele Qing, Yichuan Liu

**Affiliations:** 1 Key Laboratory of Stratigraphy and Paleontology of the Ministry of Natural Resources, Institute of Geology, Chinese Academy of Geological Sciences, Beijing, 100037 China; 2 Unaffiliated, 43125 Parma, Italy; 3 Inner Mongolia Museum of Natural History, Huhhot, 010010 Inner Mongolia China; 4 China University of Geosciences, Beijing, 100083 China

**Keywords:** Evolution, Palaeontology, Phylogenetics

## Abstract

Dromaeosaurids were bird-like dinosaurs with a predatory ecology known to forage on fish, mammals and other dinosaurs. We describe *Daurlong wangi* gen. et sp. nov., a dromaeosaurid from the Lower Cretaceous Jehol Biota of Inner Mongolia, China. Exceptional preservation in this specimen includes a large bluish layer in the abdomen which represents one of the few occurrences of intestinal remnants among non-avian dinosaurs. Phylogenetically, *Daurlong* nests among a lineage of short-armed Jehol Biota species closer to eudromaeosaurs than microraptorines. The topographic correspondence between the exceptionally preserved intestine in the more stem-ward *Scipionyx* and the remnants in the more birdlike *Daurlong* provides a phylogenetic framework for inferring intestine tract extent in other theropods lacking fossilized visceral tissues. Gastrointestinal organization results conservative among faunivorous dinosaurs, with the evolution of a bird-like alimentary canal restricted to avialan theropods.

## Introduction

Dromaeosauridae is a clade of small- to mid-sized theropod dinosaurs known from the Cretaceous of both hemispheres^1^. The Lower Cretaceous Jehol Biota from north-eastern China has provided a rich diversity of dromaeosaurids, the majority of which referred to Microraptorinae^2–7^. The affinities of two other Jehol Biota dromaeosaurids, *Tianyuraptor ostromi*^6^ and *Zhenyuanlong suni*^7^, are more problematic. These dromaeosaurids share some derived features with Microraptorinae^7^, yet they differ in having relatively shorter forelimbs and a larger body size, recalling other Laurasian dromaeosaurids (i.e., Eudromaeosauria^1^). The abundance of Jehol Biota dromaeosaurids, when not due to taxonomic oversplitting^1^, may be ecologically explained assuming niche segregation and avoidance of direct resource competition. This palaeoecological interpretation is supported by the diversity in body size, cranio-dental and appendicular specializations reported in these taxa^1–7^. Here, we describe a new dromaeosaurid from the Lower Cretaceous of the Pigeon Hill locality, Inner Mongolia (China), which shows the first case of intestinal preservation in a theropod lineage very close to bird ancestry.

Institutional abbreviation: IMMNH, Inner Mongolia Museum of Natural History, Hohhot, China.

## Results

### Systematic palaeontology

Dinosauria, Theropoda, Dromaeosauridae, *Daurlong wangi* gen. et sp. nov.

**Figure 1:**
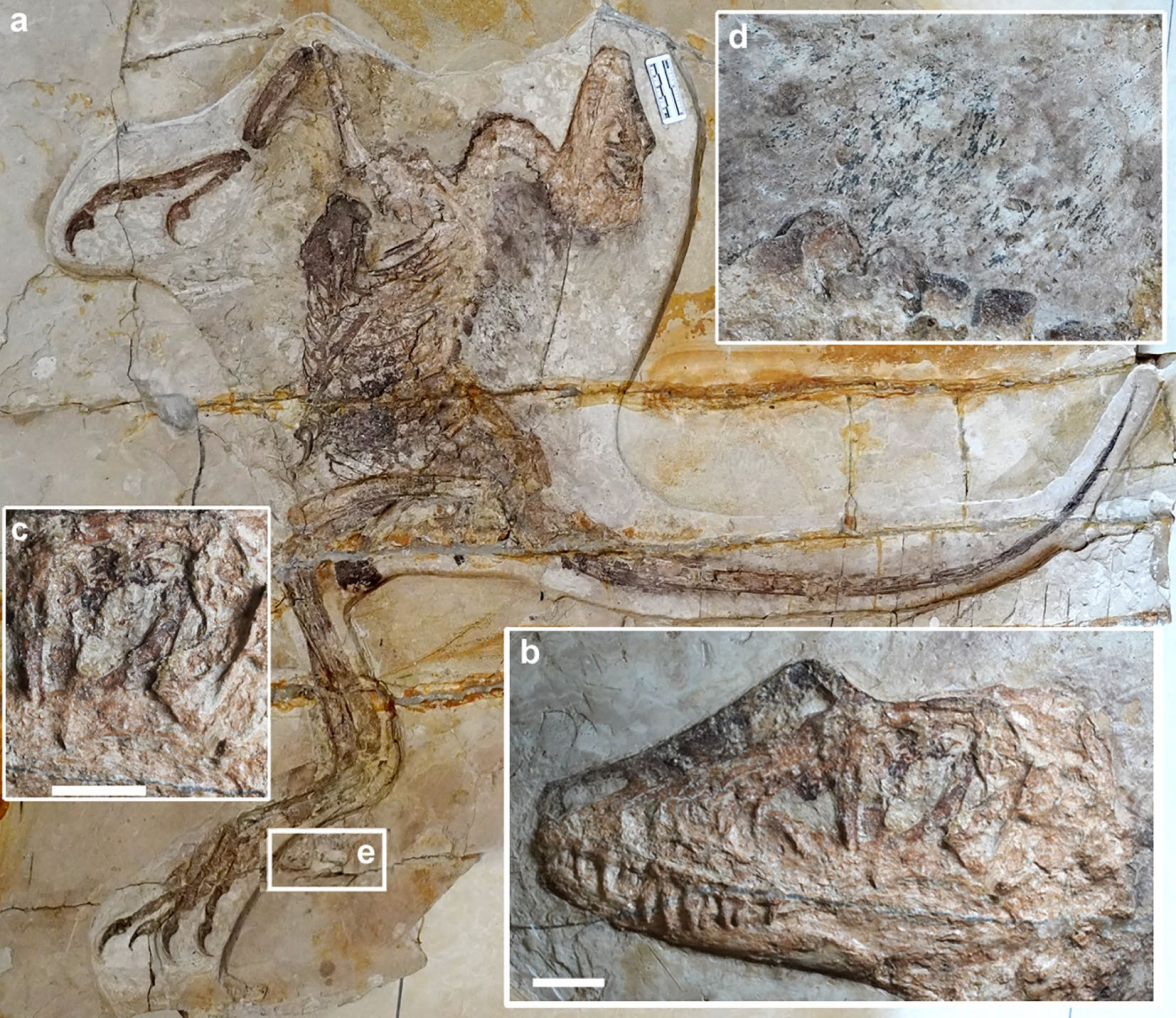
*Daurlong wangi* holotype. (**a**), whole specimen. (**b**), skull. (**c**), detail of orbit region. (**d**), feather remains associated to the thoracic vertebrae. (**e**), anuran skeleton. Scale bars: 20 mm (**b**), 10 mm (**c**).

**Figure 2:**
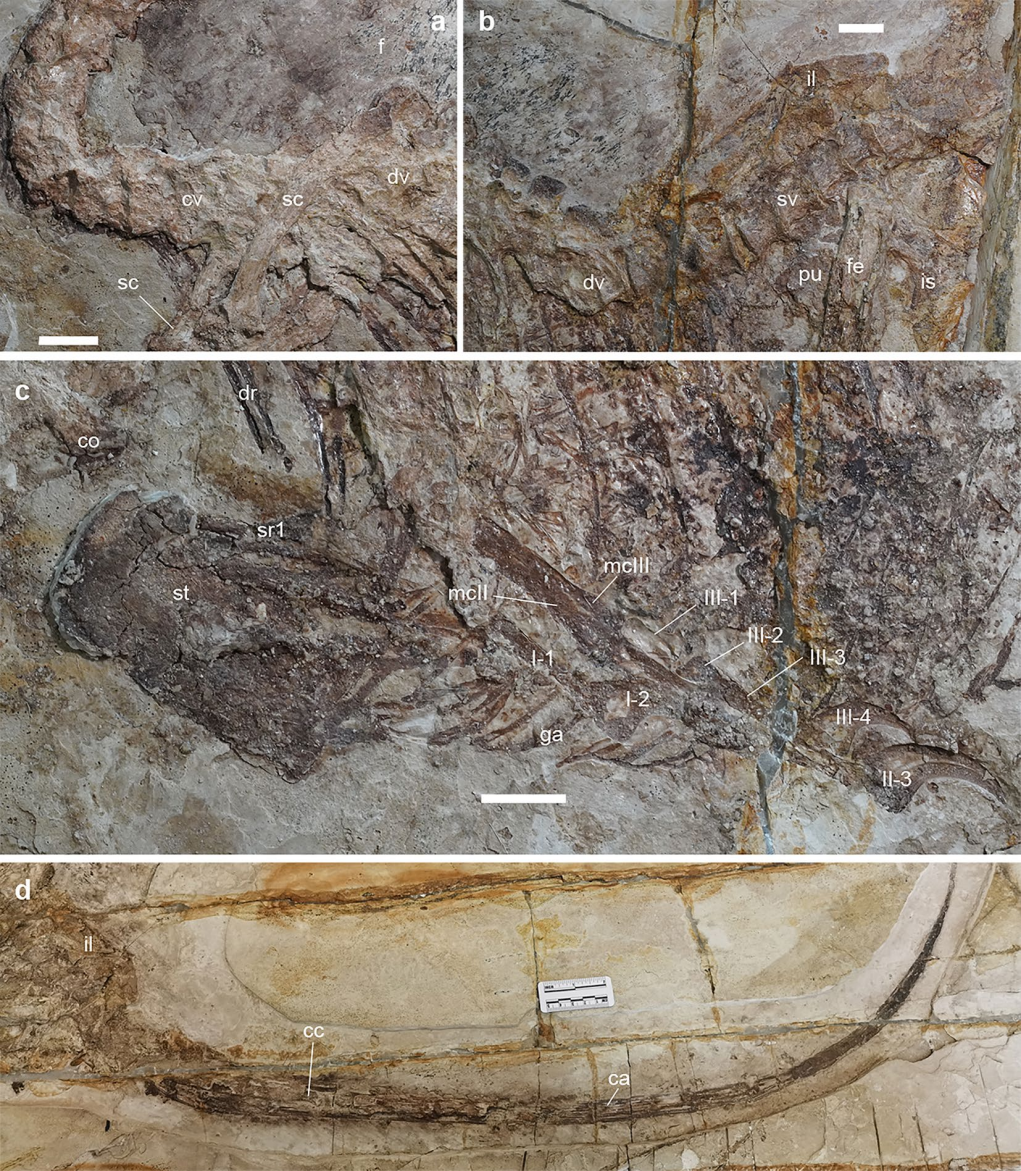
Selected elements of *Daurlong wangi* holotype. (**a**), neck and pectoral region. (**b**), thoraco-sacral series. (**c**), ventral part of the belly region. (**d**), tail. Abbreviations: ca, caudotheca; cc, caudal centrum; co, coracoid; cv, cervical vertebrae; dr, dorsal rib; dv, dorsal vertebrae; f, feathers; fe, femur; ga, gastralia; I-/II-/III-, phalanges; il, ilium; is, ischium; mc, metacarpal; pu, pubis; sc, scapula; st, sternum, sr1, sternal rib1, sv, sacral vertebrae. Scale bars in (**a-c**) = 20 mm.
- *Locality and Horizon* Pigeon Hill, Morin Dawa Daur Autonomous Banner, Inner Mongolia Autonomous Region (N 48°39′40.76″/E 123°52′ 41.15″); Longjiang Formation, Lower Cretaceous.
- *Etymology* The genus name is derived from the Daur Nation, and the Chinese 龙 ("lóng") for "dragon". The species name honors Mr. Wang Junyou, director of the IMMNH.
- *Diagnosis* Mid-sized dromaeosaurid with (autapomorphies marked by asterisk): slender subnarial ramus of premaxilla extended caudally well beyond the external naris; large, trapezoid promaxillary recess placed at the rostroventral corner of antorbital fossa*; maxillary fossa large, shallow and caudodorsally located, so that the *pila promaxillaris* is wider than the pila interfenestralis*; stepped transition from the subcutaneous surface of maxillary ventral ramus to the antorbital fossa; fan-shaped distal end of first sternal rib*. Differential diagnosis: *Daurlong* further differs from *Tianyuraptor* because it bears longer and more robust maxillary teeth and a more robust ulna. *Daurlong* further differs from *Zhenyuanlong* because it lacks a pitted ventral ramus of the antorbital fossa, lacks markedly concave distal margins in maxillary tooth crowns, bears a bowed scapula, a more robust radius, and a wider overlap of the semilunate carpal over metacarpal II (Fig. 3).

- *Holotype* IMMNH-PV00731, an almost complete dromaeosaurid (Figs. 1, 2, Supplementary information).

**Figure 3:**
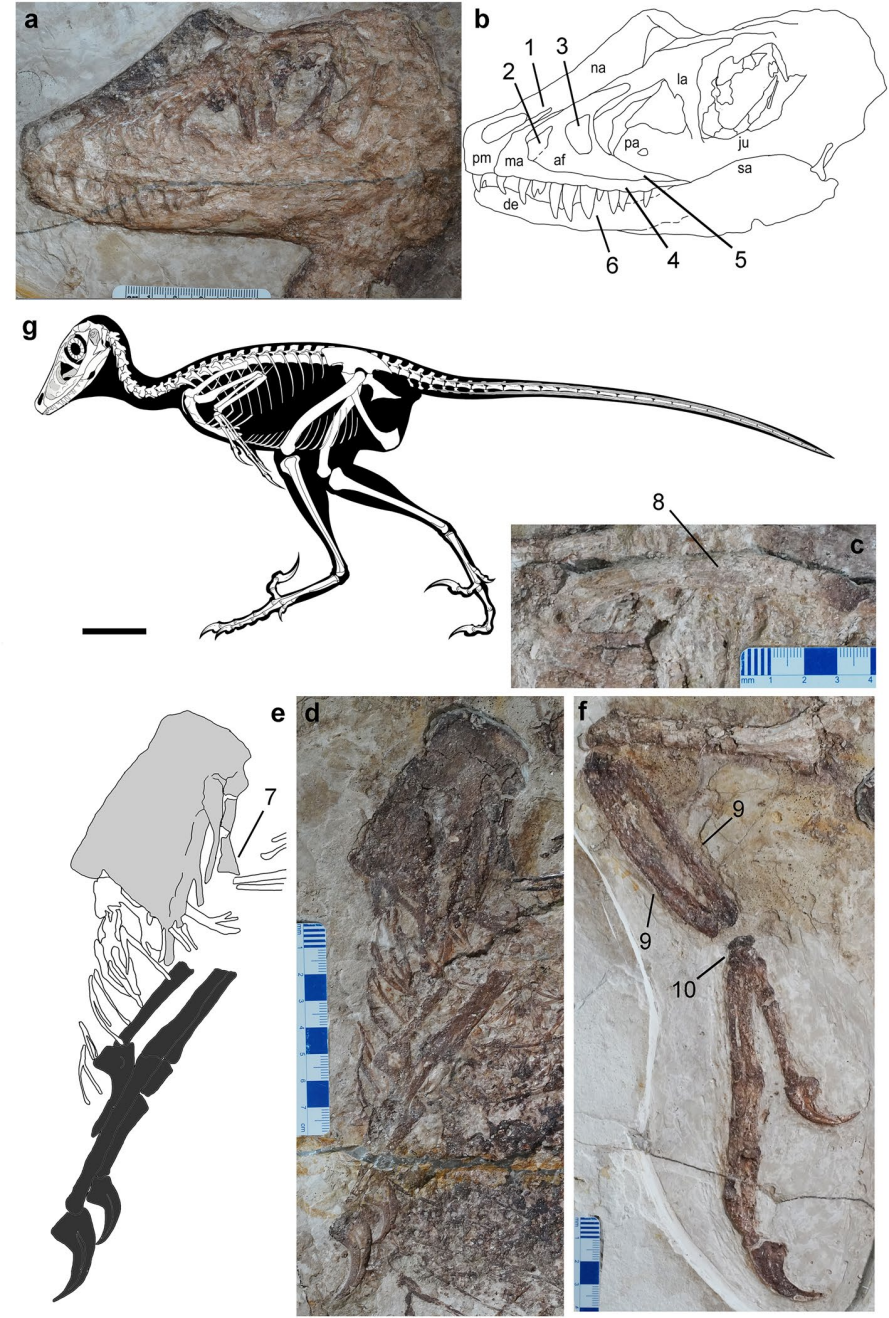
Diagnosis of *Daurlong wangi*. Specimen IMMNH-PV00731. Skull (**a**, **b**), left scapula (**c**), sternum and left hand (**d**, **e**), right forelimb (**f**). Reconstruction in (**g**) by M. Auditore (CC-BY 4.0). Combination of features diagnostic for *D. wangi*: 1, slender subnarial ramus of premaxilla extended caudally well beyond the external naris; 2, large, trapezoid promaxillary recess placed at the rostroventral corner of antorbital fossa; 3, maxillary fossa large, shallow and caudodorsally located, so that the *pila promaxillaris* is wider than the *pila interfenestralis*; 4, stepped subcutaneous surface of the ventral ramus of maxilla; 5, absence of pitted ventral ramus of the antorbital fossa; 6, robust fang-like maxillary teeth with straight to slightly convex distal crown margins; 7, distal end of first sternal rib fan-shaped. 8, bowed scapula; 9. radius and ulna more robust than any manual element; 10. wide overlap of the semilunate carpal over metacarpal II. In D, gray area indicates sternum, black areas indicate left hand elements. Abbreviations: af, antorbital fossa; de, dentary; ju, jugal; la, lacrimal; ma, maxilla; na, nasal; pm, premaxilla; su, surangular. Scale bar in G = 10 cm.

### Description and comparison

The holotype of *Daurlong wangi* is an almost complete and articulated skeleton with a length of about 150 cm (Fig. 1). Part of the ribcage and the corresponding gastral region are overlapped by the left forelimb (segments of the humerus and forearm are missing) and suffered some crushing during diagenesis. The specimen is 85% the size of *Tianyuraptor ostromi* holotype^6^, 93% the size of *Zhenyuanlong suni* holotype^7^*,* and between 115 and 350% the size of the Jehol Biota microraptorines^5,8^. The skull is almost perfectly articulated, except for the missing nasal ramus of the left premaxilla and the partially displaced dorsal parts of the right nasal and lacrimal (Figs. 1, 3). A similar displacement of nasal and lacrimal is visible in the holotype of *Zhenyuanlong*^7^. The skull is about 94% of femur length, comparable to most taxa (e.g., *Halszkaraptor*, *Microraptor* and *Saurornitholestes*) whose skulls are approximately 92–95% of femur length^8–13^. In *Zhenyuanlong*, the skull is about 86% of femur length^7^, and in *Tianyuraptor* the skull is longer than the femur^6^. In *Bambiraptor* and *Sinornithosaurus*, the skull is about 103–105% longer than the femur^11^. In *Velociraptor*, the skull is between 117 and 128% of femur length, due to the relatively elongate snout^10^. The rostral margin of the premaxilla forms a right angle with the occlusal margin, similar to *Velociraptor*, *Zhenyuanlong* and *Saurornitholestes*^2,10,12^, differing from the shallower premaxillae with acute rostroventral corner in *Microraptor* and *Sinornithosaurus*^4,11^ and the shallow platyrostral premaxilla of *Halszkaraptor*^13^. The subnarial process of the premaxilla extends caudodorsally well beyond the caudal margin of the external naris, as in *Velociraptor* and *Linheraptor*^12,14^, differing from the relatively shorter processes in *Microraptor* and *Zhenyuanlong*^2,5^*. Saurornitholestes* shows an intermediate condition*.* The external naris is relatively small, tear-shaped with the long axis directed caudodorsally, similar to *Zhenyuanlong*^7^ and differing from the relatively larger external naris in *Microraptor*^4^. In eudromaeosaurs, the external naris is relatively shorter and its long axis is subparallel to the oral margin^10,12^.

The subcutaneous part of the rostral ramus of the maxilla is relatively small and triangular, taller than long and similar to *Zhenyuanlong*^7^ and *Wulong*^15^, differing from the relatively more elongate ramus in *Halszkaraptor*^13^, *Microraptor*^4^ and in some eudromaeosaurs^14,16,17^. The antorbital fossa is large, longer than tall and covering more than two-thirds of the maxilla, similar to *Zhenyuanlong* and *Wulong*^7,15^, differing from the relatively shorter excavation seen in some eudromaeosaurs (e.g., *Acheroraptor*^17^). As in *Zhenyuanlong,* the rostral margin of the antorbital fossa is at the level of the second maxillary tooth, more rostral than in other dromaeosaurids^4,7,12^. The promaxillary fenestra is relatively large, as in other Jehol Biota dromaeosaurids^6^ and is placed adjacent to the rostroventral corner of the antorbital fossa, differing from the more caudally placed fenestra in *Sinornithosaurus* and *Zhenyuanlong*^7,11^. In *Microraptor*, the promaxillary fenestra is narrower rostrocaudally and elongated dorsoventrally^4^. The promaxillary fenestra is trapezoid in lateral view, with straight rostral, caudal and ventral margins, and a caudodorsally slanted dorsal margin. The maxillary recess is a large shallow fossa delimited by curved ridges paralleling the rostral margin of the antorbital fenestra. The *pila promaxillaris* is rostrocaudally wider than the *pila interfenestralis* (Supplementary information), a condition differentiating *Daurlong* from both *Tianyuraptor* and *Zhenyuanlong*, which show a relatively narrower *pila promaxillaris*^6,7^*.* In microraptorines^4,11^ the area around the *pila promaxillaris* and the region underneath the antorbital fenestra are excavated by a series of small pits and ridges, absent in *Daurlong*. The caudoventral ramus of the antorbital fossa lacks the pits and crests present in *Zhenyuanlong*^7^. The dorsoventrally shallow subcutaneous surface of the ventral ramus is separated from the antorbital fossa by a stepped margin, differing from *Zhenyuanlong* which shows a distinct antorbital rim^7^. As in other dromaeosaurids^17–19^, the postrantral wall is widely exposed in lateral view.

The lacrimal is T-shaped as in most dromaeosaurids^4,10–12^. The frontal widely contributes to the dorsal margin of the orbit. The orbit is ovoid-shaped with the long axis directed rostroventrally-caudodorsally (Fig. 1). The scleral ring is nearly completely preserved inside the orbit. Its dorsoventral axis coincides with that of the orbital fenestra, and does not appear significantly deformed, allowing for an accurate measurement of its inner and external diameters. The scleral ring is large (external diameter about 93% of orbital diameter) and moderately slender. The rostral ramus of the postorbital is upturned, forming an obtuse angle with the caudal ramus, which is instead perpendicular to the ventral ramus. The elements of the caudal portion of the skull are badly preserved. The dentary shows a length/depth ratio of approximately 8, proportionally intermediate between the more gracile conditions in *Microraptor* and *Velociraptor,* and the relatively stouter dentary of *Saurornitholestes*^4,10,12^. The dorsal margin is slanted rostroventrally at its rostralmost end, then, caudal to the second alveolus, it is parallel to most of the ventral margin, as in most non-dromaeosaurine dromaeosaurids^4,10^. The ventral margin of the dentary gradually tapers rostrodorsally from the rostral sixth of the bone, and then is inclined caudodorsally along the caudal third. The postdentary elements of the mandible are badly preserved.

The single premaxillary tooth crown preserved is unserrated, bears a sharp apex, and is slightly curved distally near the apex. Ten teeth are preserved in the maxilla, with an additional tooth missing. The crowns are more robust and elongate than those of *Tianyuraptor*^6^. The middle maxillary teeth are the longest and fang-like, similar to those of *Microraptor*^4^. The crowns of the second, fourth and last preserved maxillary tooth bear dense serrations along their distal carinae. All maxillary teeth are blade-shaped and only slightly curved distally near the apex, differing from the more curved teeth of *Zhenyuanlong*^7^. Six dentary teeth can be recognized. They are much smaller than the maxillary teeth. Ten cervical vertebrae are preserved in articulation, but badly preserved (Fig. 2). The cranial neural spines of the dorsal series are rectangular and strongly inclined caudodorsally. The caudal neural spines of the dorsal series are square-shaped, with their long axis oriented dorsally, almost vertical to the corresponding centra (Fig. 2). The sacrum includes six vertebrae. The sutures along the sacral centra are obliterated, except for a faint suture visible between centra 2 and 3. The first caudal vertebra is similar in size to the last sacral vertebra. The tail is complete, approximately 4.4 times longer than the femur, but the number of the vertebrae is uncertain (Fig. 2). Most of the caudal vertebrae are encased in the caudotheca^21^ as in microraptorines and eudromaeosaurs^4,20^. *Daurlong* is similar to other Jehol Biota dromaeosaurids^4,6,7^ in having the caudotheca extended through the rostralmost vertebrae, differing from eudromaeosaurs where the bony rods bundle begins distal to caudal 6^20,21^. The scapula is strap-like and uniformly bowed dorsally, differing from the straight scapula of *Zhenyuanlong*^7^. As in most paravians, the scapula is shorter than the humerus and more gracile than mid-shaft diameter of the ulna, with no distal expansion. The left coracoid is partially preserved. The bone is fused to the scapula, and both contribute to the laterally-facing glenoid, as in other paravians^1,22^. There is no evidence of the large coracoid fenestra present in some microraptorines^1,4^. The sternum is large and subrectangular with the long axis as long as the humeral shaft (Fig. 2). It appears as a single unpaired element, as in *Microraptor*^21^, and differing from the incompletely fused elements in *Halszkaraptor* and eudromaeosaurs^1,14,24^. Four pairs of sternal ribs articulate with the sternum, as in *Microraptor* and *Sinornithosaurus*, differing from *Zhongjianosaurus* which shows five pairs^25^. The first pair of sternal ribs is more robust than the others and shows an elongate fan-shaped morphology expanded laterodistally, differing from the slender shaft and the spoon-shaped distal end shared by other dromaeosaurids^5,25^. In some avialans^26^, the caudalmost sternal rib is the most robust and shows an expanded distal end similar to the condition in *Daurlong*. As in other paravians, the gastralia contact the pubis shaft in a position more proximal than in other theropods, where instead such contact is usually placed on the pubic foot^27^.

The forelimb length is less than 60% of the hindlimb. Among dromaeosaurids, comparably short forelimbs are shared with *Austroraptor*^19^, halszkaraptorines^9^, *Tianyuraptor*^6^ and *Zhenyuanlong*^7^, and differ from the relatively longer forelimbs in *Buitreraptor*^28^, microraptorines^4,5^, and some eudromaeosaurs^20^. The deltopectoral crest is not prominently developed. The ulna is slightly bowed caudally and more robust than in *Tianyuraptor*^6^. The radial shaft is about half the thickness of the ulna and nearly equal to the mid-shaft of the manual phalanx I-1: in *Zhenyuanlong* the radius is apomorphically more gracile^7^. In the carpus, the stout scapholunare articulates distally with the semilunate carpal, which, in turn, overlaps the whole extent of the proximal end of metacarpal II, differing from the more limited overlap in *Zhenyuanlong*^7^. The combined metacarpal I + manual phalanx I-1 complex is longer than metacarpal II, differing from microraptorines which have metacarpal II longer than metacarpal I + phalanx I-1^4,5^. Manual digit II is the longest and bears the largest claw. As in microraptorines and eudromaeosaurs^4,5,20^ manual phalanx III-2 is shortened, being about half the length of phalanx III-1. All the three recurved manual claws bear prominent flexor tubercles and horny sheaths. The ilium is 64% of the femoral length. The preacetabular process is longer than the postacetabular process of the ilium, a feature shared with *Halszkaraptor*, *Tianyuraptor* and *Unenlagia* among dromaeosaurids^6,9,29^, and comparable to *Rahonavis* and most avialans^30^. As in other dromaeosaurids^5^, the postacetabular process is gradually declined caudoventrally but does not extend below the level of the ischial peduncle. The pubic shaft is straight and caudoventrally oriented. The pubic foot is broadly rounded and dorsoventrally expanded, as in other Jehol Biota dromaeosaurids^2^. The ischium is about half the pubis in length, lacks dorsal processes along the straight dorsal margin and ends distally in a poorly curved tip. The obturator process is triangular, as long as deep, and placed at mid-lenght of the ventral margin, differing from the prominent and more distally-placed process in microraptorines^5^.

The femur is 91% of the tibia in length (in both *Tianyuraptor* and *Zhenyuanlong*, the femur is < 77% of the tibia). In theropods, the femur-to-tibia ratio is allometrically controlled^31^, and results higher in large-bodied dromaeosaurids than in small-bodied taxa^32^. The feet are incomplete, with the medialmost elements (i.e., toes I and II) badly preserved and overlapped by other foot bones. Metatarsal III and IV are nearly equal in length and about 61% of the femoral length. The preserved four pedal claws are comparable in size, are strongly recurved and bear low but prominent flexor tubercles.

The plumage is preserved along the dorsal margin of the postorbital part of the skull, adjacent to the presacral neural spines (Fig. 1d) and along the edges of the tail. No feathers are preserved close to the limbs or along the ventral margin of the body: it is unclear if long pennaceous remiges and rectrices were present as in *Zhenyuanlong*^7^. The plumage along the dorsal margin of the pre-caudal part of the skeleton is preserved as a series of compound structures containing several filaments joined at their proximal ends, similar to the condition in *Sinornithosaurus*^33^. Short pennaceous feathers are preserved along most of the margin of the caudotheca. The tail feathers appear symmetrical, oriented backward and forming a low angle (~ 15–20°) with the proximodistal axis of the adjacent tail vertebrae. Scanning electron microscopy (SEM) of sampled portions of the tegumentary remains failed to identify melanosomes^34–36^. The caudal half of the abdominal cavity of the theropod is occupied by a black-bluish layer which is bound by the gastral basket (ventrally) and the pubis (caudally) (Figs. 1, 2, Supplementary Information). The rostral margin of the layer is placed ventral to the 9th dorsal centrum, where it extends dorsoventrally along the dorsal half of the abdominal cavity. The black-bluish layer reaches its maximum depth between the 10th and the 11th dorsal vertebrae, where it reaches the ventralmost part of the gastral basket. Caudal to the 11th dorsal vertebra, the layer is limited to the dorsal end of the abdominal cavity.

The imprint of the partial skeleton of an anuran is preserved in the same slab of IMMNH-PV00731, adjacent to the theropod metatarsi (Fig. 1).

## Discussion

### Phylogenetic affinities of *Daurlong*

**Figure 4:**
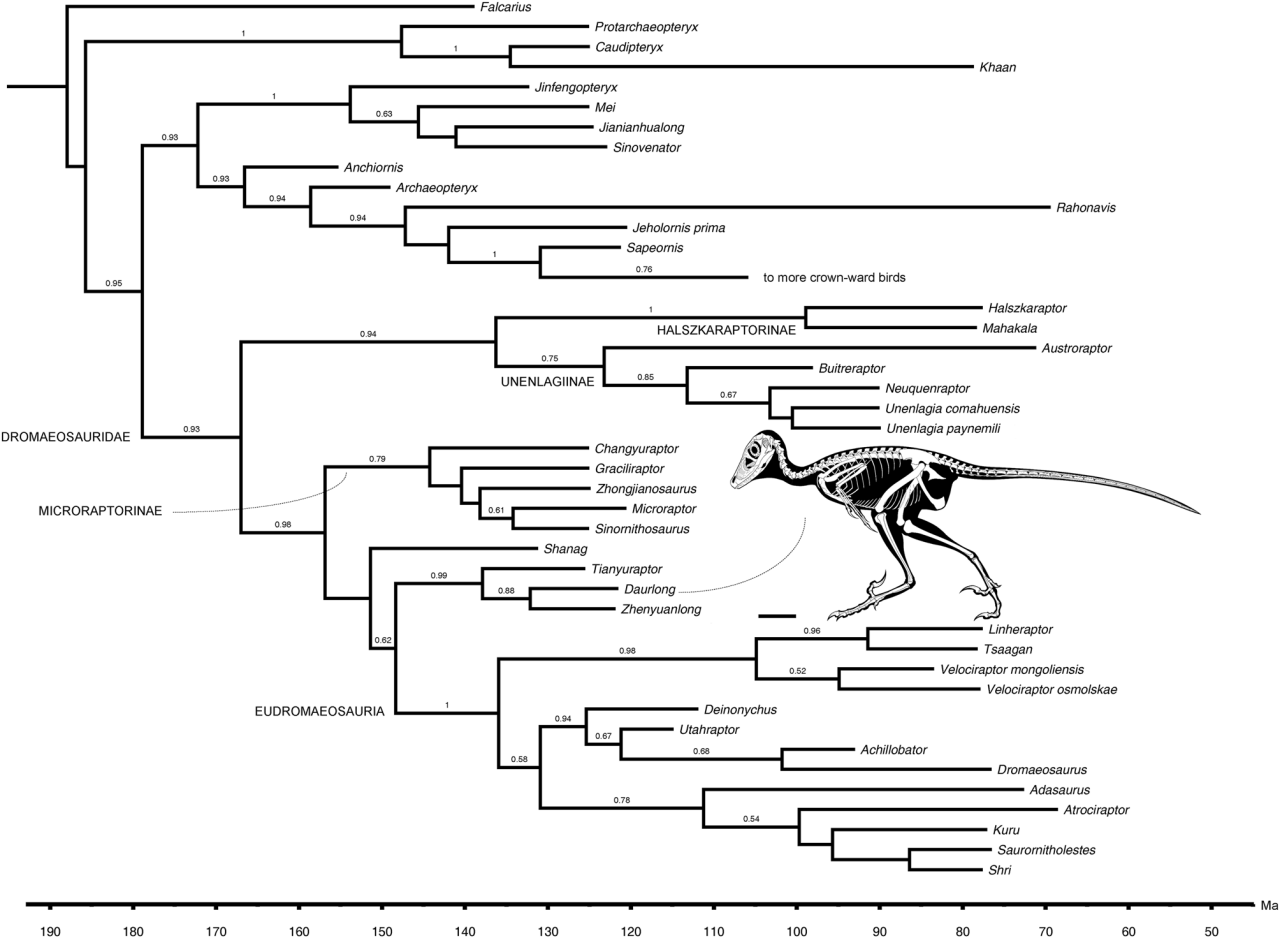
Affinities of *Daurlong wangi*. Time-calibrated Maximum Clade Credibility Tree reconstructed by the tip-dating Bayesian inference analysis. Values at branches indicate posterior probability. Scale bar = 100 mm. Skeletal drawing credit: Marco Auditore (CC-BY 4.0).

*Daurlong wangi* is unambiguously referred to Dromaeosauridae based on the combination of large promaxillary recess^4,11^, caudodorsally placed maxillary recess^4,11,12^, tail with caudotheca^21^, shortened manual phalanx III-2^4–7^ and caudoventrally-oriented pubis with cup-shaped distal foot^2–7^. It shows the closest phyletic affinity with *Tianyuraptor* and *Zhenjuanlong*^6,7^ (Fig. 4; Supplementary Information). In particular, *Daurlong* shares with these dromaeosaurids the antorbital region which is very extensive rostrally (resulting in a very short subcutaneous part of the preantorbital maxilla), a reduced deltopectoral crest of the humerus, and the elongation of the preacetabular process of the ilium^6,7^. These three dromaeosaurids are significantly larger in body size than the other Jehol Biota paravians (e.g.,^3,4^). *Daurlong* differs from *Tianyuraptor* and *Zhenyuanlong* in the absence of accessory antorbital pits, in the robustness and curvature of the maxillary teeth, and in the relative robustness of the forearm elements. The relationships between the "*Tianyuraptor*-like" clade and their closest relatives are weakly-supported. This is likely due to the mosaic morphology of the "*Tianyuraptor*-like" taxa which combines features alternatively supporting close relationships with Microraptorinae or with Eudromaeosauria^1,6^. It is noteworthy that the "*Tianyuraptor*-like" taxa retain features ancestral to both the above mentioned clades, shared with earlier-diverging dromaeosaurids (i.e., halszkaraptorines and unenlagiines) or with non-dromaeosaurid paravians (e.g., troodontids), such as the absence of a fossa bounding the maxillary recess^4,18^, the relatively short forelimbs (i.e., shared with troodontids, halszkaraptorines and some unenlagiines^9,19^), and the elongation of the preacetabular process of the ilium (i.e., shared with the halszkaraptorines, some unenlagiines, and avialans^9,19^).

### Conservatism in non-avian theropod gastrointestinal organization

The reconstruction of the gastrointestinal track in extinct taxa, including dinosaurs, could be inferred, indirectly, from gut content remains; less frequently by the analysis of coprolite contents; and rarely from exceptionally preserved remnants of the soft tissues^37–48^. The topographic distribution of the bluish layer in the caudal half of the ribcage in IMMNH-PV00731 (Fig. 1, Supplementary Information) closely matches the extent of the intestinal track preserved in the non-avian theropod *Scipionyx*^41^. As in *Scipionyx* intestine, the bluish layer does not extend cranial to the 9^th^ dorsal vertebra and shows its maximum dorsoventral extent at the level of the 10^th^-12^th^ dorsal vertebrae, where it reaches the gastral basket. In both cases, an “empty” region separates the intestinal mass from the cranial margin of the pubis shaft^41^. At the micrometric scale, the bluish layer is formed by a fabric of densely packed microcrystals ranging 1–3 micron in diameter (Supplementary information), as in *Scipionyx* intestine^41^, suggesting that it represents the product of authigenic mineralisation^49^ driven by the activity of decay bacteria, which replicated the large-scale outline of the decayed distal part of the gastrointestinal track^41^. Intestinal record in non-avialan theropods is rare, occurring in *Scipionyx*^41^ and *Mirischia*^50^. The close topographic correspondence between the intestinal tracks in *Daurlong* and *Scipionyx* relative to their axial skeletons might provide the basis for inferring the extent of the distal digestive region in other theropods bracketed phylogenetically by these taxa. Ovoid structures in the abdominal cavity of a specimen of *Sinosauropteryx*, and argued to be eggs^42^, closely match in shape and position the duodenal portion of *Scipionyx* intestine^41^ and the ventral part of the bluish layer in *Daurlong*, and are here re-interpreted as intestinal remnants. The re-evaluation of the purported “eggs” of *Sinosauropteryx* as intestinal portions is more in agreement with the skeletal immaturity of the specimen^42^ and dismisses an adult status for this compsognathid-grade theropod^51^. From an evolutionary perspective, the topographic correspondence between the whole intestinal mass in *Scipionyx* (considered an early-diverging coelurosaur^41^ or alternatively an allosauroid^51^) and the paravian *Daurlong* supports conservatism in intestinal general organization among faunivorous theropods. The evolution of a bird-like alimentary canal is thus inferred as an avialan innovation^46^. As in *Scipionyx*, the presence of intestinal remnants in *Daurlong* contrasts with the complete absence of any remnant of the stomach. Distinct taphonomic patterns, regulated by the physiological pH of the decaying organ, finely tuned the authigenic mineralisation: this process might had been inhibited by the persistence of the extremely acid environment of the stomach immediately after the death of the animal^41,49^.

## Methods

### UV fluorescence photography

In order to identify any fluorescing minerals (e.g., calcium phosphate deposits, which discriminate preserved periosteal surfaces from eroded bones), the specimen was illuminated with a short wave UV lamp.

### Scanning electron microscopy

Scanning electron microscopy (SEM) was obtained on samples removed from IMMNH-PV00731 with sterile dental tools, placed on carbon tape on copper stubs, sputter coated with Au and examined using a JEOL JSM-6700 and a ZEISS SIGMA-500 SEM at accelerating voltages of 15–20 kV**.**

### Phylogenetic analyses

The phylogenetic affinities of the new dromaeosaurid were investigated scoring a taxonomic operational unit (OTU) based on IMMNH-PV00731 in a data set focusing on basal paravian and dromaeosaurid relationships. Character statement list was based on the data set of^13^ expanded with novel character statements described by^18^ (Supplementary information). Parsimony analysis was performed in TNT 1.5^52^ performing 100 'New Technology' analyses followed by exploration of the sampled island using the 'Traditional Search' analysis. Nodal support was calculated sampling 10.000 trees up to ten steps longer than the shortest topologies (Supplementary information). Bayesian inference analyses integrating morphological and stratigraphic data were performed in BEAST 2.6^53,54^, implemented with the packages for the analysis of morphological characters, using the model of^55^, and for sampling potential ancestors among the ingroup^56^. In our analysis, rate variation across traits was modeled using the multi-gamma parameter (implemented for the analysis of morphological data in BEAST 2). The rate variation across branches was modeled using the relaxed log-normal clock model, with the number of discrete rate categories that approximate the rate distribution set as n − 1 (with n the number of branches), the mean clock rate using default setting, and not setting to normalize the average rate. Since the character matrix includes autapomorphies of the sampled taxa, the model was not conditioned to variable characters only. Stratigraphic information for the taxa was converted to geochronological ages. Stratigraphic data and age constraints for each terminal were obtained from the Paleobiology Database (http:// paleobiodb.org/), checked against the International Chronostratigraphic Chart (http:// stratigraphy. org/), and included as uniform priors for tip-dating. The extant taxon included (the avian *Meleagris*) calibrates the height for the tip-date setting (the uniform prior setting used for incorporating uncertainty in the age of the fossil taxa requires at least one terminal taxon to have the tip age fixed to a value^56^). The analysis used one replicate run of 100 million generations, with sampling every 1000 generations. In the analyses, burnin was set at 40%. Convergence and effective sample sizes of every numerical parameter among the different analyses were identified using Tracer^53,54^. The root age of the tree model was conservatively set as a uniform prior spanning between the age of the oldest in-group taxa and 200 Mya (near the Triassic-Jurassic boundary), which consistently pre-dates the diversification of all maniraptoran branches.

### Nomenclatural act

This published work and the nomenclatural acts it contains have been registered in ZooBank, the online registration system for the ICZN. LSIDurn:lsid:zoobank.org:pub:9C845D24-3CC2-47CD-8993-E946C2D27D15.

- *Daurlong*: LSIDurn:lsid:zoobank.org:act:28B9C1F5-665F-4517-81C1-25F7066D61F8.
- *Daurlong wangi*: LSIDurn:lsid:zoobank.org:act:61,938,885–1854-4CCC-B723-C4065C83BEBA.

## Supplementary Information

Supplementary Information 1.

Supplementary Information 2.

Supplementary Information 3.

## Data Availability

All data generated or analysed during this study are included in this published article and its supplementary information files.
