# Supplementary material for: Intestinal preservation in a birdlike dinosaur supports conservatism in digestive canal evolution among theropods
Source: Sci Rep. 2022 Nov 19;12:19965. doi: 10.1038/s41598-022-24602-x (PMC9675785; doi:10.1038/s41598-022-24602-x)
Supplement: Supplementary file 1 — Supplementary Information 1. [file 41598_2022_24602_MOESM1_ESM.pdf]

SUPPLEMENTARY INFORMATION to

Intestinal preservation in a birdlike dinosaur supports conservatism in digestive canal evolution among theropods

Xuri Wang*, Key Laboratory of Stratigraphy and Paleontology of the Ministry of Natural Resources, Institute of Geology, Chinese Academy of Geological Sciences, Beijing 100037, China; 147966459@qq.com

Andrea Cau*, Unaffiliated, 43125, Parma, Italy; cauand@gmail.com

Bin Guo, Inner Mongolia Museum of Natural History, Huhhot 010010, Inner Mongolia, China; 594924646@qq.com

Feimin Ma, Inner Mongolia Museum of Natural History, Huhhot 010010, Inner Mongolia, China; mafeimin2005@163.com

Gele Qing, Inner Mongolia Museum of Natural History, Huhhot 010010, Inner Mongolia, China; qinggele1025@163.com

Yichuan Liu, China University of Geosciences, Beijing 100083, China; 1976570361@qq.com

*corresponding authors

Institutional abbreviations

IMMNH, Inner Mongolia Museum of Natural History, China.

Table S1. Measurements of *Daurlong wangi* holotype IMMNH-PV00731

	mm
skull length	170
skull height at orbit	85
orbit length (measured along craniocaudal axis)	44
scleral ring inner diameter (measured along vertical axis)	27.5
scleral ring outer diameter (measured along vertical axis)	41
last premaxillary crown apicobasal diameter vs mesiodistal base diameter	5 x 2.5
1st maxillary crown apicobasal diameter	6
2nd maxillary crown apicobasal diameter	9.6
4th maxillary crown apicobasal diameter vs mesiodistal basal diameter	13.5 x 4.5
6th maxillary crown apicobasal diameter vs mesiodistal basal diameter	13.5 x 4.5
7th maxillary crown apicobasal diameter	3
tail length	800
scapula length	112
scapula neck width	10
sternum length	75
humerus length	120
ulna length	96
radius length	90
metacarpal I length	20
manual digit I-1 length	36
manual digit I-2 length	30
metacarpal II length	50
manual digit II-1 length	30
manual digit II-2 length	43
manual digit II-3 length	37
metacarpal III length	48
manual digit III-1 length	17
manual digit III-2 length	10
manual digit III-3 length	20
manual digit III-4 length	17
ilium length	115 ^{estimated}
preacetabular process of ilium length	40 ^{estimated}
postacetabular process length	33
pubis length	147^{estimated}
ischium length	92^{estimated}
femur length	180
tibia length	198
metatarsal III length	110
pedal digit III-1 length	35
pedal digit III-2 length	30
pedal digit III-3 length	23
pedal digit III-4 length	30
metatarsal IV length	95
pedal digit IV-1 length	30
pedal digit IV-2 length	20
pedal digit IV-3 length	20
pedal digit IV-4 length	13
pedal digit IV-5 length	25

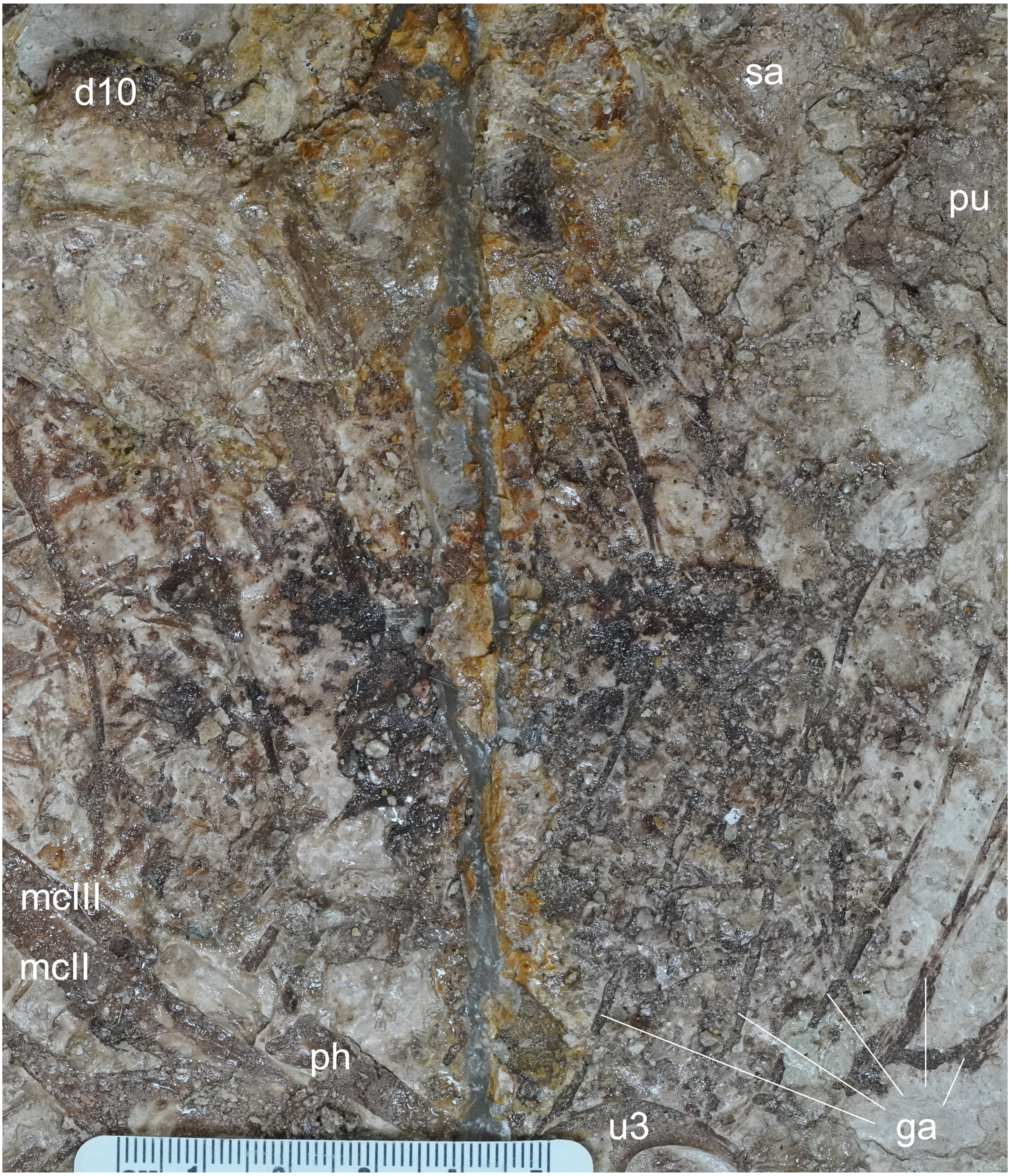

Figure S1. Detail of the abdominal region in *Daurlong* skeleton IMMNH-PV00731 showing the distribution of the bluish layer. Abbreviations: d10, 10th dorsal neural spine; ga, gastralia; mcII, metacarpal II; mcIII, metacarpal III; ph, manual phalanx; pu, proximal end of pubis; sa, sacrum; u3, 3rd manual ungual. Scale bar: 50 mm.

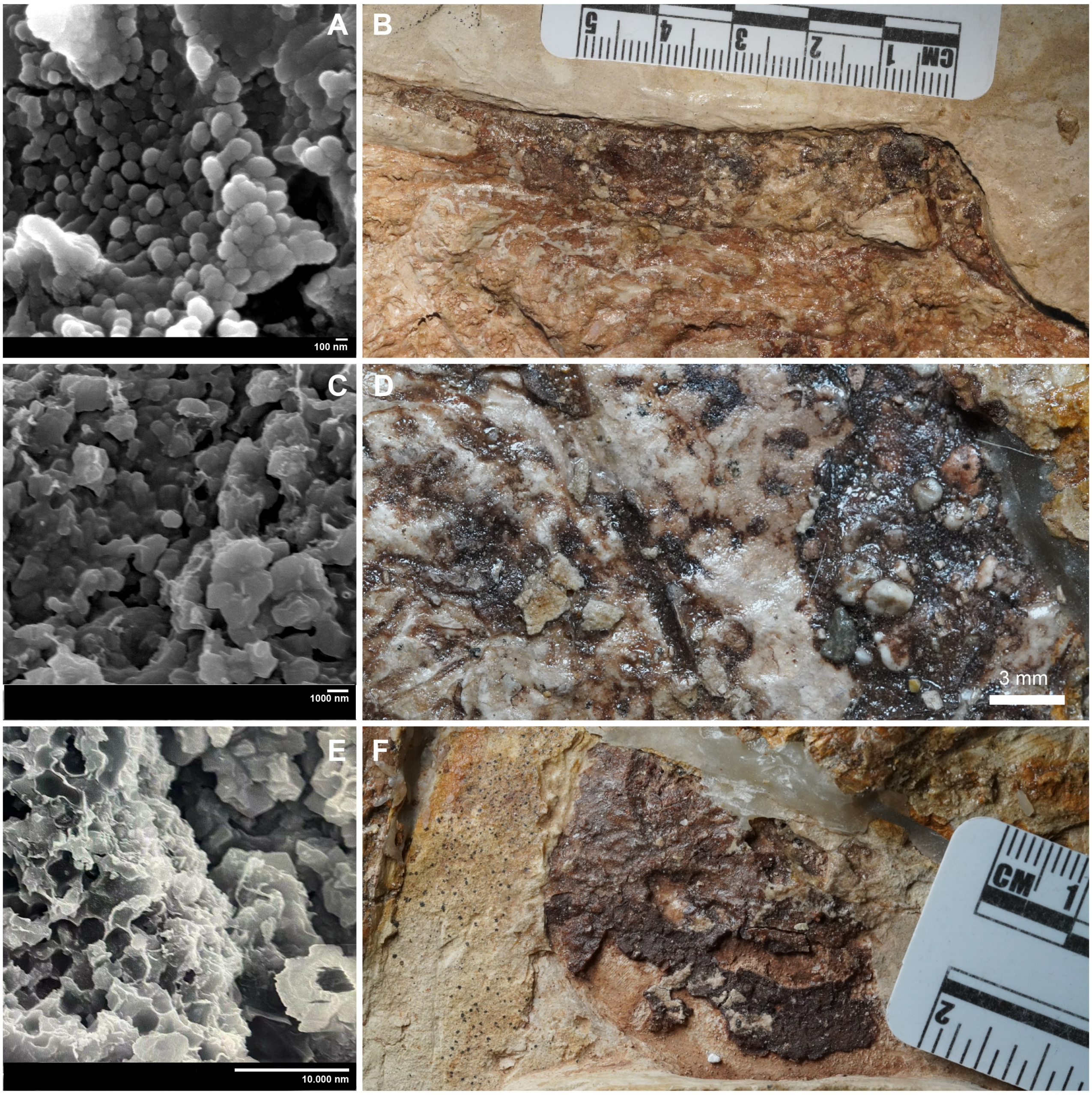

Figure S2. SEM of selected samples of the brown-blue areas of IMMNH-PV00731 dismissing integumentary preservation. A, B, nasal. C, D, blueish layer in the abdominal region. E, F, pubic foot. Note in SEM images (A, C, E) the abundance of microcrystals and framboids but the absence of melanosomes.

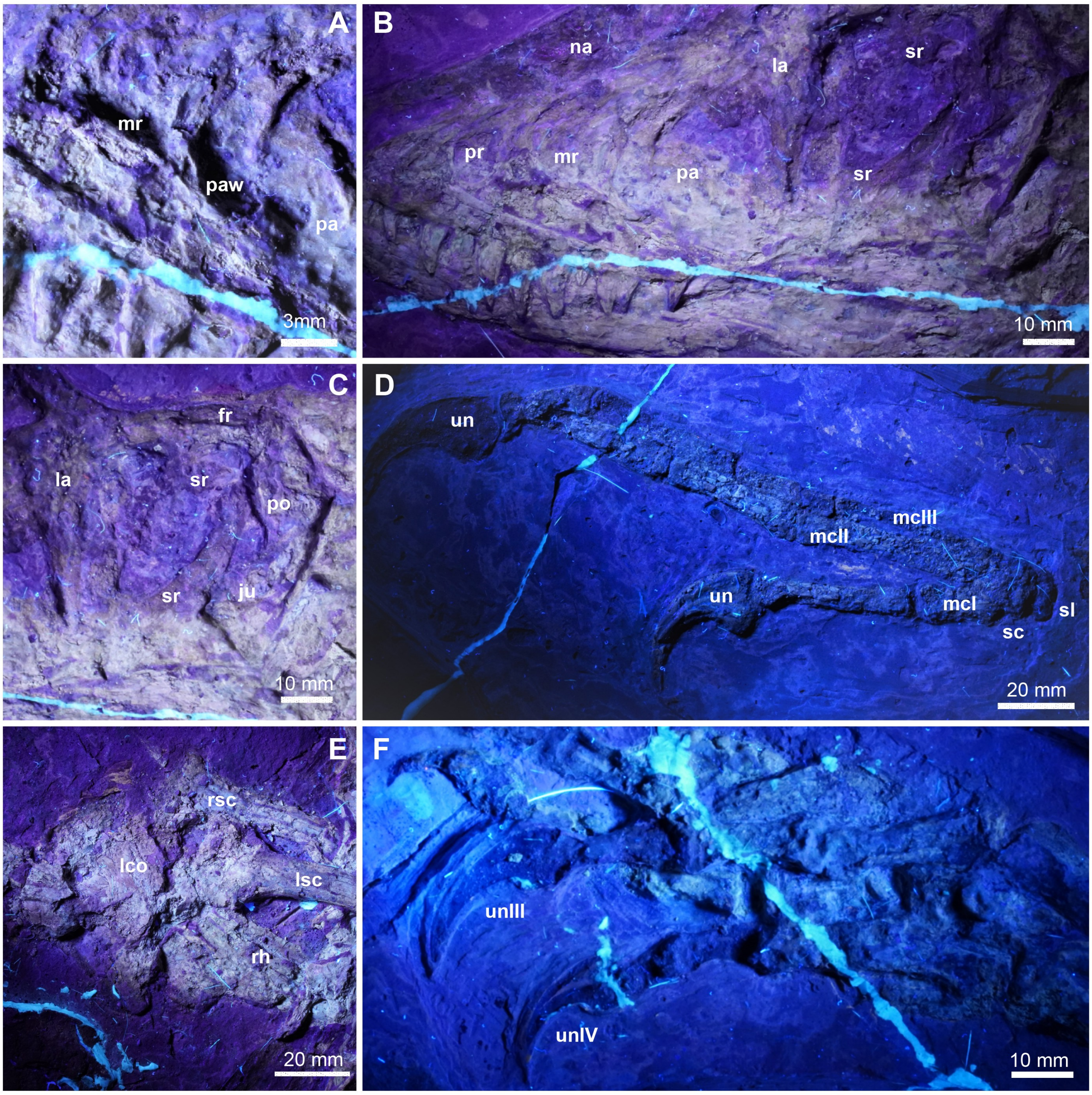

Figure S3. UV fluorescent photographs of selected regions of IMMNH-PV00731. A, close up of the antorbital fossa. B, detail of the skull. C, detail of the orbital region. D, right hand. E, pectoral region. F, feet. Fluorescence is limited to the areas appearing white/pale under white light (see Figures 1,2), confirming them as zones of periosteal preservation. Abbreviations: fr, frontal; la, lacrimal; lco, left coracoid; lsc, left scapula; ju, jugal; mc, metacarpal; mr, maxillary recess; na, nasal; pa, palatine; paw, postantral wall; po, postorbital; pr, promaxillary recess; rh, right humerus; rsc, right scapula; sc, semilunate carpal; sl, scapholunare; sr, sclerotic ring; un, ungual.

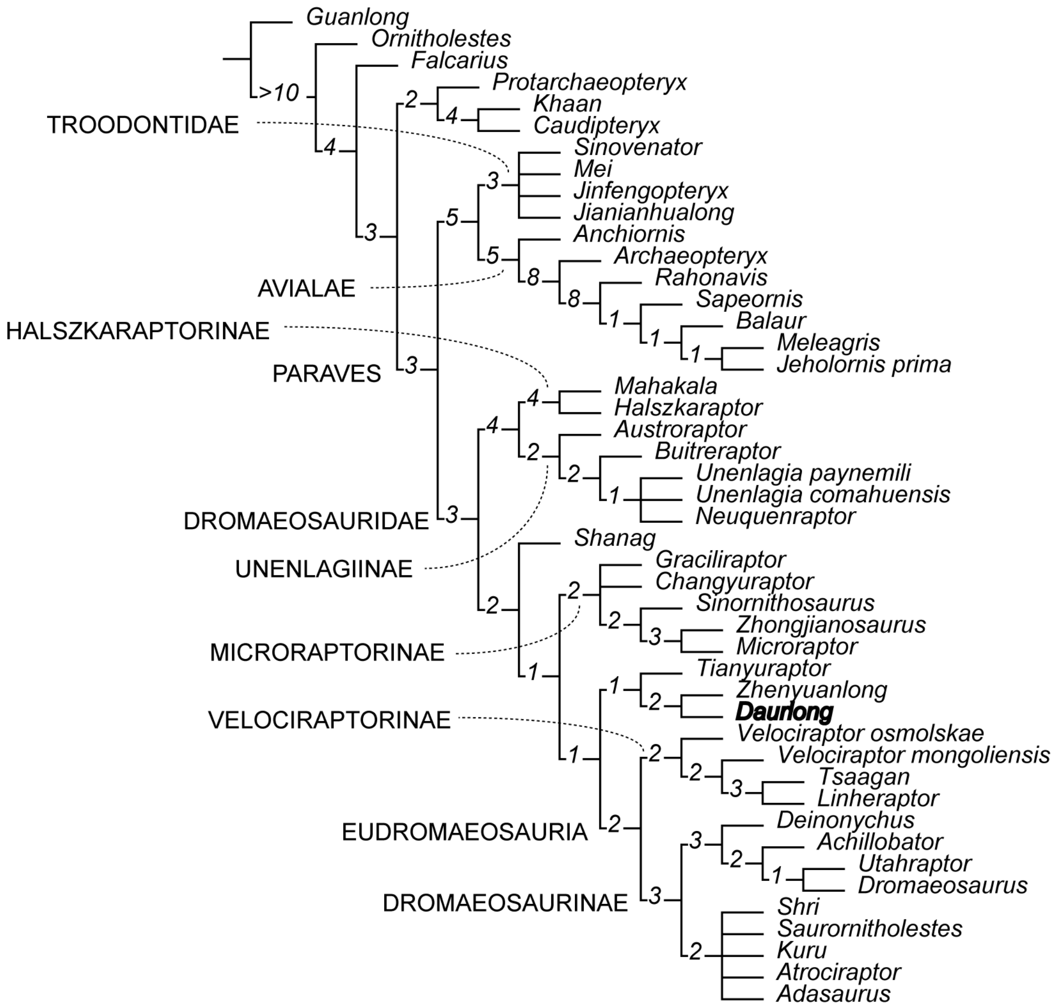

Figure S4. Strict consensus of the 12 shortest trees reconstructed by the parsimony analysis. Values at nodes indicate Bremer support. Tree length = 2154. Consistency Index excluding uninformative characters = 0.3559. Retention Index = 0.4637.

Phylogenetic analysis

Character list

Character statements 1-1807 based on [13]: https://doi.org/10.7717/peerj.8672/supp-1

New character statements (1808-1864):

1808): Caudal vertebrae, accessory longitudinal ridge running along dorsolateral surface of neural arch, lateral to zygapophyses and medial to ribs: absent (0); present (1).

1809): Caudal vertebrae, anterior and middle neural arches, posterior view, fossa on peduncle, ventrolateral to poszygapophyseal bases: absent (0); present (1).

1810): Anterior caudal vertebrae, prespinal lamina, development: poorly- to moderately-developed, not markedly protruding anteriorly in lateral view (0); prominent, forming a distinct convex anterior margin of the neural spine at mid-height (1).

1811): Femur, shaft, posterior surface, area medial to fourth trochanter, fossa: absent (0); present, elliptical to triangular in shape (1).

1812): Feathers, forelimb, pennaceous layers, number: one (0); more than one (1).

1813): Feathers, forelimb, pennaceous layers, differentiation: absent, all feathers of similar size and shape (0); distinction between shorter coverts and longer remiges (1).

1814): Radiale and intermedium: distinct (0); fused into a single bone (=scapholunar) contacting proximally both radius and ulna (1).

1815): Pterygoid, vomerine contact, development: well-developed (0); small process overlapping palatine (1).

1816): Pterygoid-vomer contact: present (0); absent (1).

1817): Series of scutate scales along mid-line ventral surface of tail: absent (0); present (1).

1818): Manual non-ungual phalanges, shaft, lateral and medial sides, longitudinal furrow: absent or shallow, variably developed in the phalanges (0); present, deep and distinct in all phalanges (1).

1819): Basioccipital apron, basisphenoid overlap, proximodistal extent: less (0); more (1) than 1/3 of the basisphenoid.

1820): Basisphenoid, collateral scars placed lateral to middle depression, elliptical fossae: absent (0); present and distinct on both sides (1).

1821): Premaxilla, narial margin, overlap with oral margin: present (0); absent (1).

1822): Premaxilla, interalveolar space between 3rd and 4th alveoli, mesiodistal diameter: less (0); subequal or more (1) than ½ of 4th alveolus mesiodistal diameter.

1823): Frontal, postorbital facet, orientation in dorsal/ventral view relative to anteroposterior axis of bone: lateral to anterolateral (0); posterolateral (1).

1824): Sacral series in taxa with more than four sacral vertebrae, ventral surface, shape in lateral view: straight (0); curved (ventrally concave) (1).

1825): Femur, fourth trochanter, orientation in posterior view: posteriorly, not approaching the medial margin of shaft (0); posteromedially, approaching the medial margin of shaft (1).
1826): Tibia, lateral cnemial crest, medial surface, proximodistally-oriented crest: absent (0); present (1).
1827): Metatarsal III, shaft, posterior surface, distal end, step-like eminence: poorly developed (0); well-defined as a rugose platform (1).
1828): Mesencephalon and optic lobes, position: mostly exposed dorsally (0); shifted lateroventrally relative to dorsal surface of cerebrum and cerebellum (1).
1829): Cerebellum-cerebrum dorsal contact: absent (0); present (1).
1830): Nasal, subnarial ramus, length: less (0); more (1) than dorsal (main) body of nasal.
1831): Maxilla, maxillary recess, medial wall, perforation, extent: not complete (foramen into depressed fossa) (0); complete (whole recess is fenestrated). Inapplicable in taxa lacking a medial perforation of the recess.
1832): Sternum, paired posteromedial processes (placed in the middle of the posterior margin of each plate): absent (0); present (1).
1833): Pedal ungual III, length: less (0); more (1) than 2/5 of metatarsal III.
1834): Metatarsal II, extensor surface, tubercle for M. tibialis cranialis, size: small tubercle (0); large boss (1).
1835): Coracoid, lateral margin, extent of the convex margin: limited to the distal third (0); extended more proximally (1). (Inapplicable in taxa lacking a lateral convexity).
1836): Sternum, posterolateral process, distal expansion, shape in ventral/dorsal view: symmetrically expanded relative to process long axis (0); asymmetrically expanded, lateral corner more obtuse and medial corner more acute (1). (Inapplicable in taxa lacking a distally expanded lateral trabecula).
1837): Fibula: longer (0); shorter (1) than half of tibia.
1838): Dentary, occlusal margin in adult, extent: more (0); less (1) than half of mandible (symphysis-glenoid distance).
1839): Pubis-gastralia ligament, proximodistal position on pubis: distalmost end (pubic foot, when present) (0); more proximal (to pubic foot, when present) (1).
1840): Pubis-gastralia ligament, anteroposterior position on pubis: lateral margin (0); anterior margin (1).
1841): Tibia, proximal end, posterior margin in lateral/medial view: straight (0); anteriorly bent (1) approaching the proximal condyles.
1842): Dorsal vertebrae, anterior view, triangular spinoprezygodiapophyseal fossa: absent (0); present and bound ventrally by an accessory lamina (1).

1843): Caudal vertebrae, anterior and middle centra, pleurocentral depression: absent (0); present (as shallow oval fossa or true pleurocoel penetrating centrum) (1).
1844): Manual ungual I, collateral sulci, proximal end, exposition: completely exposed (0); partially to fully invaginated/enclosed by bone (1).
1845): Metatarsal IV, proximal shaft, anterior margin, cross section, shape: flat to convex (0); describing a sharp ridge (1).
1846): Metatarsal IV, distal end, accessory pit placed on the lateral half of the extensor surface and separated from the lateral collateral fossa by a thick ridge: absent (0); present (1).
1847): Metatarsal IV, distal shaft width: uniform along proximodistal axis (0); narrowing (1).
1848): Caudal vertebrae, middle prezygapophyses, dorsal surface, longitudinal sulcus: absent (0); present (1).
1849): Tibia, shaft in anterior/posterior view: straight (0); bowed (concave medially) (1).
1850): Tibia, proximal end, posterior surface distal to condylar region, elliptical rugose scar: absent (0); present (1).
1851): Metatarsal IV, shaft, distal end, cross section: quadrangular, dorsally flat or broadly convex (0); triangular, dorsally narrower (1).
1852): Pedal phalanx P1-IV, dorsal surface, mediolateral diameter relative to ventral surface: not significantly compressed, gradual transition from lateral and medial surfaces (0); compressed, resulting in sharp medial and lateral keels bordering the dorsal surface (1). (Baiano et al., 2022).
1853): Pedal phalanx P1-IV, extensor pit, position: central (0); laterally-placed (1).
1854): Metatarsal IV, mid-shaf width: more (0); less (1) than half distal end width.
1855): Caudal vertebrae, prezygocostal lamina, anterior end, thickened tuberosity ("accessory transverse process"): absent (0); present (1).
1856): Axis, spinopostzygapophyseal laminae, development: low ridges (0); prominent (1).
1857): Scapula, glenoid facet, anteroposterior orientation relative to long anteroposterior axis of bone: perpendicular (0); forming an acute angle directed posteroventrally (1).
1858): Fronto-nasal suture, shape in dorsal view: simple curve (0); complex, interdigitating (1).
1859): Parasphenoid, subsellar recess, development and exposition in anterior view: shallow, poorly visible (0); deep and widely exposed anteriorly (1).
1860): Cheek teeth in adult, serration, denticle number along each carina: less (0); more (1) than 250.
1861): Maxilla, maxillary recess, posterodorsal corner, relationships with antorbital fenestra: separated (0); confluent (1).
1862): Maxilla, maxillary recess, fossa placed posterodorsal to maxillary fenestra, additional foramen: absent (0); present (1). Inapplicable in taxa lacking a combination of maxillary fenestra

inside a larger fossa.

1863): Dorsal vertebrae in adult, middle and posterior neural spines, dorsoventral diameter relative to anterior centrum dorsoventral diameter: less (0); more (1) than three times.

1864): Caudotheca (hyperelongate chevrons and postzygapophyses), anterior extent: distal to (0); proximal to (1) the sixth caudal vertebra. Inapplicable in taxa lacking the caudotheca.
